# Supplementary material for: Biodistribution of nanoplastics in mice: advancing analytical techniques using metal-doped plastics
Source: Commun Biol. 2025 Aug 19;8:1247. doi: 10.1038/s42003-025-08709-1 (PMC12365251; doi:10.1038/s42003-025-08709-1)
Supplement: Supplementary file 2 — Supporting Information [file 42003_2025_8709_MOESM2_ESM.pdf]

Supporting Information

Biodistribution of Nanoplastics in Mice: Advancing Analytical Techniques Using Metal-doped Plastics

Theresa Staufer[+,*,1], Verena Kopatz[+,*,2,3,4,5], Alice Pradel[6], Tess Brodie[7], Robert Kuhrwahl[1], Deborah Stroka[7], Julia Wallner[2], Lukas Kenner*[2,4,5,8,9,10] Verena Pichler[4,11], Florian Grüner[1], Denise M. Mitrano[6]

1 University of Hamburg, Fachbereich Physik, and Center for Free-Electron Laser Science (CFEL), 22761, Hamburg, Germany
2 Medical University of Vienna, Department of Pathology, Vienna, Austria
3 Medical University of Vienna, Department for Radiation Oncology, Vienna, Austria
4 CBmed GmbH – Center for Biomarker Research in Medicine, Graz, Austria
5 CCC – Comprehensive Cancer Center, Vienna, Austria
6 ETH Zurich, Environmental Systems Science Department, Zürich, Switzerland
7 University of Bern, Department of Visceral Surgery and Medicine, Inselspital, Bern University Hospital, Bern, Switzerland
8 University of Veterinary Medicine, Unit of Laboratory Animal Pathology, Vienna, Austria
9 Umeå University, Department of Molecular Biology, Umeå, Sweden
10 Medical University Vienna, Christian Doppler Laboratory for Applied Metabolomics, Vienna, Austria
11 University of Vienna, Department of Pharmaceutical Sciences, Division of Pharmaceutical Chemistry, Vienna, Austria

[+]shared first authorship between Theresa Staufer and Verena Kopatz

* **Corresponding authors**: Theresa Staufer (theresa.staufer@uni-hamburg.de), Verena Kopatz (verena.kopatz@meduniwien.ac.at), Lukas Kenner (lukas.kenner@meduniwien.ac.at)

Table S1: Summary of the samples scanned *via* XFI and reconstructed palladium masses or detection limits for the acute exposure experiment using an undiluted suspension of plastic particles for gavage and different sampling time points. The given masses are reconstructed from *one* nominal detector while the detection limits are obtained by combining the measurements from all ten detectors. The limits represent the maximum mass inside a sample which does not result in a measurable signal at any scan position in the used XFI-setup (noted as "below detection limit" = bdl in the table).

**Mouse ID / Sample name**	**Treatment time [min]**	**Total mass of Pd in sample [ng]**	**Detection limit [ng/sample]**	**Recovery [% daily dose/mouse]**
Gavage Mix (100 µL)		9000		
Stool	30	bdl		
Stool	60	bdl		
Stool	120	6		0.03
Stool	180	827		4.14
Stool	240	6030		30.23
28671 Blood (100 µL)	240	bdl	14.4 – 19.6	
28693 Stomach #	10	5560		55.70
28714 Stomach #	30	5660		56.69
28716 Stomach #	60	7130		71.49
28668 Stomach #	120	1710		17.17
28671 Stomach #	240	762		7.61
28693 Small intestine #	10	4030		40.35
28714 Small intestine #	30	6880		68.93
28716 Small intestine #	60	2910		29.12
28668 Small intestine #	120	5160		51.70
28671 Small intestine #	240	991		9.93
28693 Colon #	10	bdl		
28714 Colon #	30	bdl		
28716 Colon #	60	bdl		
28668 Colon #	120	389		3.90
28671 Colon #	240	1980		19.81
28671 Brain *	240	bdl	17.8 – 18.5	
28671 Liver ~	240	bdl	8.4 – 11.2	
28671 Kidney *	240	bdl	6.4 – 8.9	
* half of organ measured, ~ part of organ measured; # GI organs not flushed/with content				

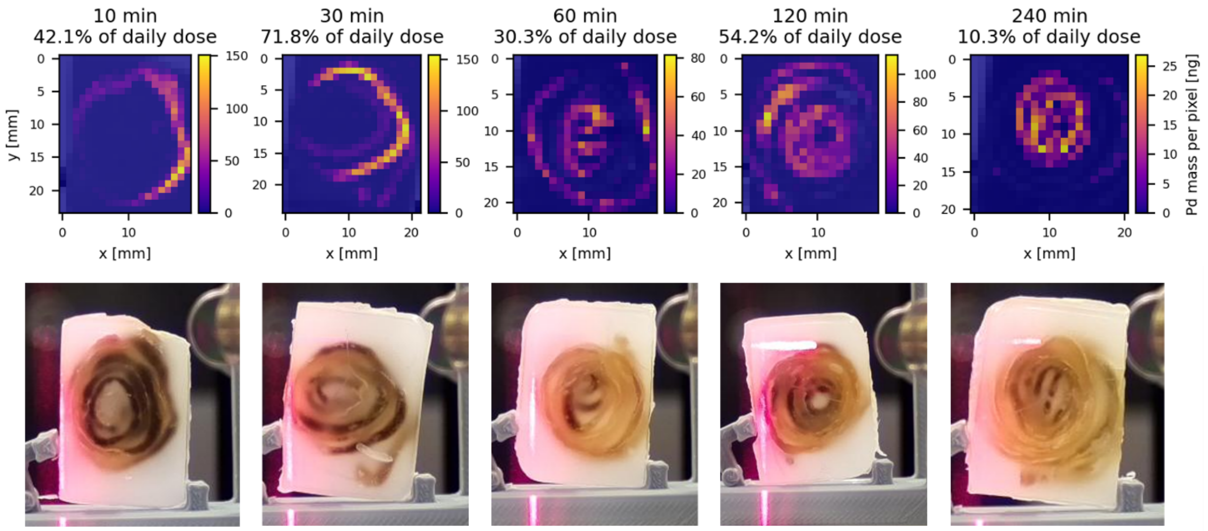

Figure S1: Direct comparison of reconstructed Pd distributions (upper row) to photographs of measured small intestine samples (bottom row) in the XFI setup.

Table S2: Pd content in samples (ng) from the gastrointestinal tract (unflushed) after certain timepoints during the acute exposure experiment as measured *via* ICP-MS. Total recovery (given as % of daily dose/mouse = applied dose) is given cumulatively for all measured GI organs.

Mouse ID	Time (minutes)	Stomach #	Small intestine #	Cecum #	Colon #	Recovery [% daily dose/mouse]
28692	10	8320.2	1542.3	17.7	2.1	70.07
28715	30	1128.0	7238.4	11.1	0.5	83.98
28717	60	749.4	6228.3	0.6	bdl	70.01
28667	120	1131.9	554.1	4887.4	2052.2	86.46
28670	240	871.9	154.5	1644.2	1713.5	47.95
# GI organs not flushed/with content, bdl = below detection limit						

Table S3: Pd content in the gavage solution and in the feces samples collected after certain timepoints during the acute exposure experiment as measured via ICP-MS. Feces from two individuals were collected and the mass of Pd as well as the recovery (% daily dose/mouse = applied dose) is given.

Stool sample timepoint	Mass of Pd in sample [ng]	Recovery [% daily dose/mouse]
Gavage Mix (100 µL)	9976	
30 min	0.19	0.001
60 min	0.19	0.001
120 min	17.9	0.09
180 min	204	1.02
240 min	6130	30.69

Table S4: Pd content in blood and organ samples (ng) other than the gastrointestinal tract for the acute exposure experiment as measured with ICP-MS.

Mouse ID	Time (min)	Blood (100 µL)	Pancreas *	Brain */Liver ~/Heart * Spleen */Kidney */ Testes *	Mesothelium
28692	10	bdl	2.6	bdl	bdl
28693	10	bdl	15.8	bdl	
28714	30	28.8	2.8	bdl	
28715	30	bdl	bdl	bdl	bdl
28716	60	bdl	bdl	bdl	
28717	60	bdl	2.2	bdl	bdl
28667	120	bdl	bdl	bdl	bdl
28668	120	bdl	bdl	bdl	
28670	240	bdl	bdl	bdl	bdl
28671	240	bdl	16.6	bdl	
* half of organ measured, ~ part of organ measured; bdl = below detection limit					

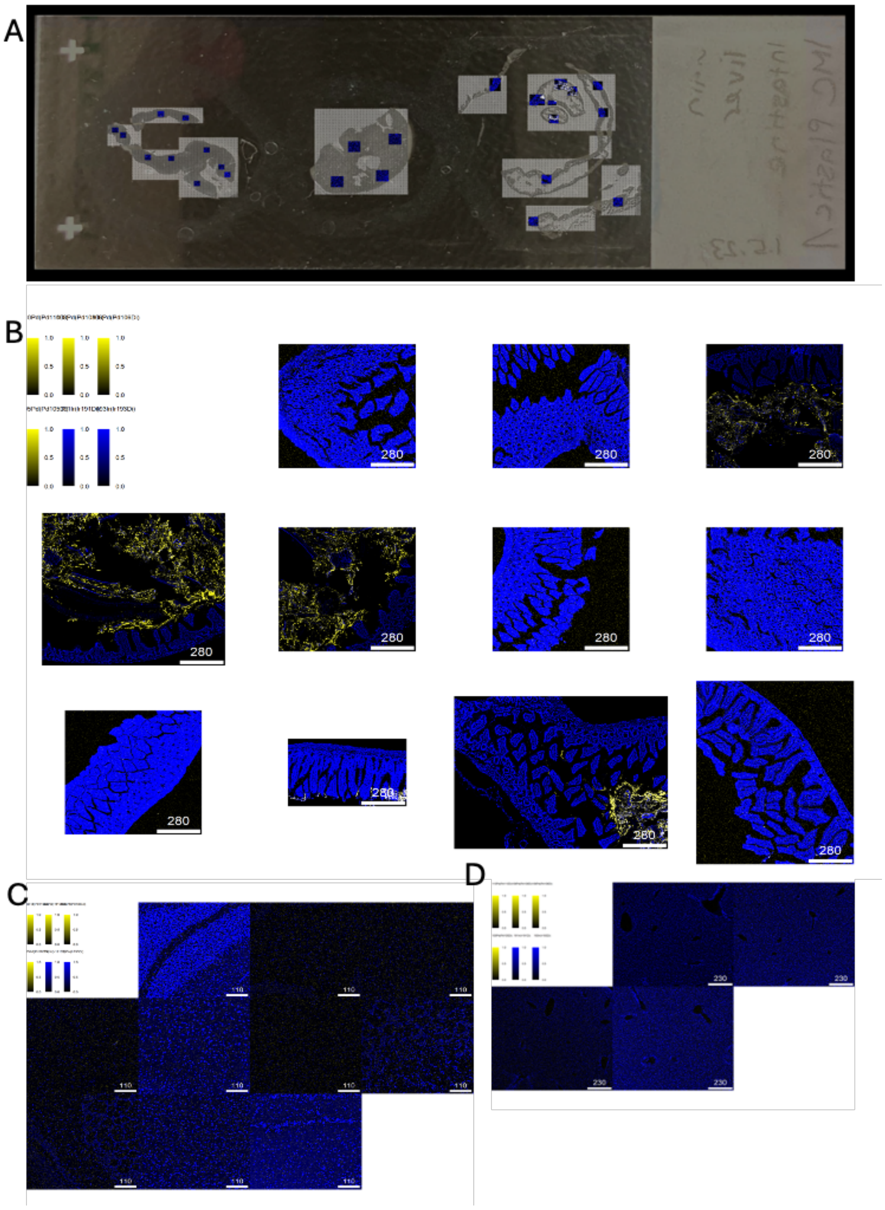

Figure S2: IMC analysis of the acute exposure experiment from mouse #28671 (240 min timepoint). (A) Slide overview photo with panorama high resolution images from the CyTOF software in light grey and laser ablated ROI with DNA in blue (image from napari) from the brain, liver and small intestine. Respective ROI images of small intestine (B), brain (C) and liver (D) of the measured areas are shown. DNA is labelled in blue and all Pd isotopes in yellow.

Table S5: Summary of the samples scanned *via* XFI and reconstructed palladium masses or detection limits for the subacute exposure experiment. The given masses are reconstructed from *one* nominal detector while the detection limits are obtained by combining the measurements from all ten detectors. The limits represent the maximum mass inside a sample which does not result in a measurable signal at any scan position in the used XFI-setup (noted as "below detection limit" = bdl in the table).

Mouse ID / Sample name	Total mass of Pd in sample [ng]	Detection limit [ng/sample]	Recovery [% daily dose/mouse]
Gavage Mix	2910		
28334 Urine (100 µL)	bdl	11.6 - 16.4	
Stool, Pd-NPs Day 9	9050		103.67
28334 Blood (100 µL)	bdl	13.9 - 16.3	
28334 Small intestine ~	bdl	35.2 - 36.6	
28334 Colon ~	bdl	10.5 - 13.1	
28334 Brain *	bdl	11.8 - 14.0	
28334 Thymus*	bdl	2.2 - 3.9	
28334 Liver ~	bdl	6.3 - 7.4	
28334 Pancreas *	bdl	9.6 - 10.9	
28334 Heart *	bdl	10.7 - 14.6	
28334 Spleen *	bdl	5.1 - 7.3	
28334 Kidney *	bdl	8.8 - 10.2	
28334 Testes *	bdl	3.2 - 3.6	
* half of organ measured, ~ part of organ measured			

Table S6: Pd content in feces (24 h sampled from 3 individuals) for the subacute exposure experiment as measured *via* ICP-MS. Values for control and Pd-NPs group in total mass (ng) per sample and recalculated as % of daily dose/mouse.

Sample name	Mass of Pd in total sample [ng]	Recovery [% daily dose/mouse]
Stool, Ctrl Day 1	201.6	2.31
Stool, Ctrl Day 3	50.2	0.57
Stool, Ctrl Day 5	3.2	0.04
Stool, Ctrl Day 9	4.6	0.05
Stool, Pd-NPs Day 1	339.4	3.89
Stool, Pd-NPs Day 3	2666.0	30.5
Stool, Pd-NPs Day 5	5417.2	62.05
Stool, Pd-NPs Day 9	7221.1	82.72

Table S7: Pd content in sample (ng) for the subacute exposure experiment from mouse 28334 (DSS + Pd-NPs) as measured *via* ICP-MS. In the case of half/part organs the recovery of % daily dose was extrapolated for the total organ under the assumption that Pd was equally distributed within the organ.

Organ	Total mass of Pd in sample [ng]	Recovery [% daily dose/mouse]
Urine (100 µL)	0.9	
Blood (100 µL)	1.9	
Small intestine ~	114.8	32.80
Colon ~	5.5	2.83
Brain *	1.4°	0.08
Thymus *	33.9	0.82
Liver ~	7.7	1.24
Pancreas *	bdl	bdl
Heart *	1.4°	0.06
Spleen *	bdl	bdl
Kidney *	1.4°	0.06
Testes *	4.8	0.20
* half of organ measured, ~ part of organ measured, bdl = below detection limit<br>° Pd content below LOQ; concentration set to LOD (1.4 ng for organs)		

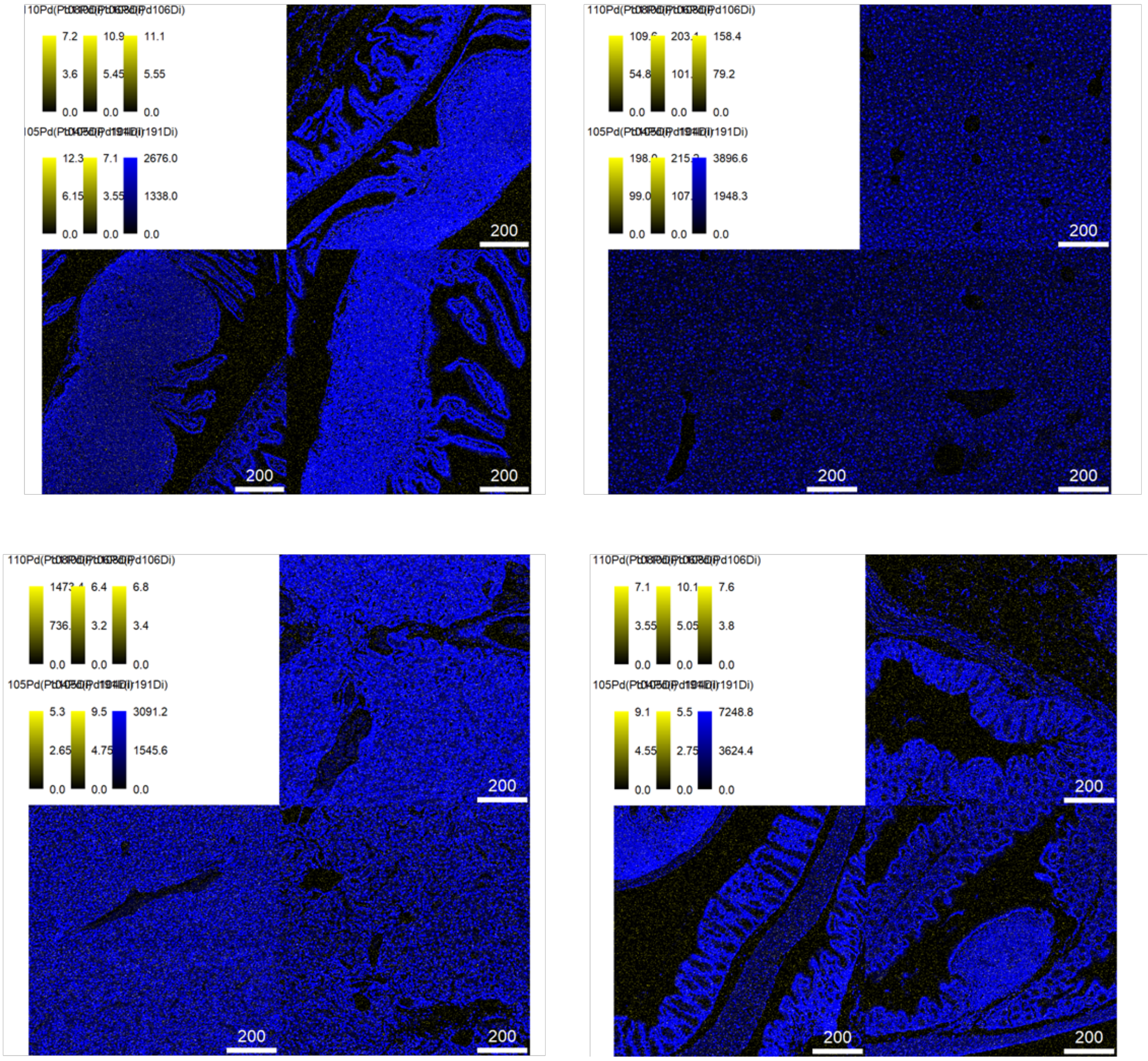

Figure S3: IMC images from the subacute exposure experiment for 4 tissue types and 3 conditions. In the top row from left to right, colon and liver, and in the bottom row from left to right, kidney and small intestine. The 3 mice used in each of the 4 tissues shown are as follows: top tissue next to the legend is Control (28328), bottom left under the legend is NP (mouse #28337), bottom right is NP + DSS (mouse #28334). DNA in blue and all isotopes of Pd in yellow.

Table S8: Summary of the samples scanned *via* XFI and reconstructed palladium masses or detection limits for the subchronic exposure experiment. The given masses are reconstructed from *one* nominal detector while the detection limits are obtained by combining the measurements from all ten detectors. The limits (LOD) represent the maximum mass inside a sample which does not result in a measurable signal at any scan position in the used XFI-setup (noted as "below detection limit" = bdl in the table).

Mouse ID / Sample name	Reconstructed Palladium mass [ng]	LOD [ng]	Recovery [% daily dose/mouse]
Stool, wildtype, 1 h	bdl		
Stool, wildtype, 2 h	0.85		0.03
Stool, wildtype, 3 h	230		8.16
Stool, wildtype, 4 h	360		12.76
Stool, wildtype, 5 h	351		12.43
Stool, wildtype 6 h	44.3		1.62
Stool, wildtype 8 h	82.6		2.93
Stool, wildtype 9 h	55.7		1.98
Stool, wildtype 10 h	177		6.28
29194 Blood	bdl	11.0 - 16.5	
29199 Blood	bdl	10.6 - 14.5	
29200 Blood	bdl	14.6 - 16.6	
29206 Blood	bdl	9.8 - 12.2	
29207 Blood	bdl	6.7 - 12.4	
29194 Brain	bdl	4.0 - 5.5	
29194 Kidney	bdl	3.0 - 3.6	
29207 Kidney	bdl	3.1 - 5.9	

Table S9: Pd content in feces for up to 10 h (sampled from 2 wildtype or 3 APCmin+ individuals) for the subchronic exposure experiment as measured with ICP-MS. Values for wildtype and APCmin+ mice in total mass (ng) per sample and recalculated as % of daily dose/mouse.

Sample name	Mass of Pd in total feces [ng]	Recovery [% daily dose/mouse]
Stool, wildtype 1 h	35.1	1.24
Stool, wildtype 2 h	10.5	0.37
Stool, wildtype 3 h	253.9	9.00
Stool, wildtype 4 h	171.9	6.09
Stool, wildtype 5 h	89.3	3.17
Stool, wildtype 6 h	59.7	2.12
Stool, wildtype 8 h	112.5	3.99
Stool, wildtype 9 h	76.0	2.69
Stool, wildtype 10 h	28.7	1.02
Stool, APCmin+ 2 h	8.4	0.20
Stool, APCmin+ 3 h	105.9	2.50
Stool, APCmin+ 4 h	200.8	4.75
Stool, APCmin+ 6 h	248.4	5.87
Stool, APCmin+ 7 h	53.7	1.27
Stool, APCmin+ 8 h	81.0	1.91
Stool, APCmin+ 9 h	398.3	9.42
Stool, APCmin+ 10 h	242.3	5.73

Table S10: Pd content (ng) in blood, gastrointestinal tract and organ tissues for the subchronic exposure experiment as measured with ICP-MS.

Mouse ID / Sample name	wildtype		APCmin+		
	29194	29199	29200	29206	29207
Blood (100 µL)	bdl	bdl	bdl	bdl	bdl
Small Intestine		10.3			1.0
Colon		4.5			bdl
Brain *	34.8	bdl	0.7°	8.7	0.7°
Liver ~	6.2	4.6	140.5	bdl	3.8
Mesothelium		13.2			9.7
Pancreas *	0.7°	10.9	0.7°	15.8	bdl
Heart *	32.8	10.6	bdl	2.0	6.2
Spleen *	2.5	0.7°	6.6	4.3	2.6
Kidney *	83.1	8.0	6.0	12.2	21.0
Testes *	10.1	15.7	8.7	bdl	bdl

* half of organ measured, ~ part of organ measured, bdl = below detection limit
° Pd content below LOQ; concentration set to LOD (0.7 ng for organs)

Table S11: Pd content as a % of daily dose, in blood, gastrointestinal tract, and organ tissues for the subchronic exposure experiment as measured with ICP-MS. In the case of half/part organs the recovery was extrapolated for the total organ under the assumption that Pd was equally distributed within the organ.

**Mouse ID / Sample name**	**wildtype**		**APCmin+**		
	**29194**	**29199**	**29200**	**29206**	**29207**
Blood (100 µL)	bdl	bdl	bdl	bdl	bdl
Small Intestine		0.73			0.07
Colon		0.32			bdl
Brain *	4.72	bdl	0.11	11.32	0.10
Liver ~	2.84	1.75	44.65	bdl	1.25
Mesothelium		0.94			0.69
Pancreas *	0.09	1.61	0.11	2.61	bdl
Heart *	3.98	1.64	bdl	0.29	0.73
Spleen *	0.32	0.07	1.21	0.97	0.42
Kidney *	11.36	1.25	0.96	1.75	2.32
Testes *	1.38	2.30	1.27	bdl	bdl

* half of organ measured, ~ part of organ measured, bdl = below detection limit

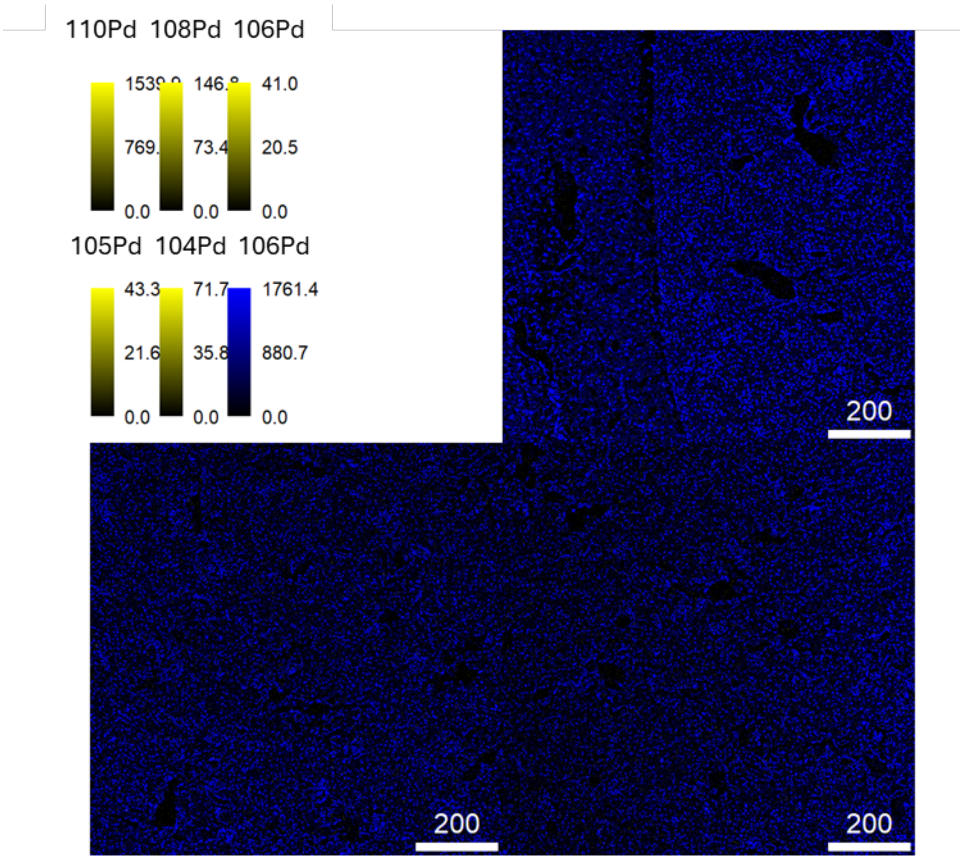

Figure S4: IMC measurements for the subchronic exposure experiment. 3 kidney ROIs from mouse #29194 (wildtype) with DNA in blue and all isotopes of Pd in yellow. The top row is ROI 1 and the bottom row from left to right is ROI 2 and 3.

Table S12: Comparison of the analytical merits of the three methods employed in this study

Method	Limit of detection (LOD) & quantification (LOQ)	Spatial resolution	Measurement interferences	Reproducibility	Time investment	Access to equipment
XFI	2.2 - 36.6 ng/sample<br><br>Full list see Tables S1, S3 and S5, as the detection limit strongly depends on the object of investigation	Defined by beam diameter; here 1 mm but can be much lower (nm-scale at dedicated synchrotron beamlines)	None	High<br><br>From all our synchrotron-based XFI measurements we have determined a reproducibility of 5% in terms of the fluctuations in counts of the measured photons. At the beginning of each measurement day we perform a photon flux reconstruction, typically with errors below 5%.	18 min for full-body scan of a mouse in vivo; scan time can vary depending on the required spatial resolution and size of the object of investigation.	Synchrotron for high-sensitivity measurements is needed, but compact, much less expensive systems are under development.<br><br>Labeling of particles but no other sample preparation needed.
ICP-MS	LOD: 0.014 - 0.019 µg/L<br>LOQ: 0.043 - 0.056 µg/L (instrumental)<br>LOD: 0.14 - 0.30 µg/L<br>LOQ: 0.43 - 0.88 µg/L (blood samples)<br>LOD: 0.42 - 1.43 µg/L<br>LOQ: 1.29 - 4.20 µg/L (organ samples)<br>LOD: 0.19 - 0.43 µg/L<br>LOQ: 0.56 - 1.26 µg/L (feces samples)	None: Spatial resolution is generated by dissecting and separating different organs	None<br>(but depending on elemental distribution of the sample, spectral interferences may be possible in ICP-MS in some cases but often can be corrected for)	Measurements of digested samples are highly reproducible. However, reproducibility can vary due to the sample preparation requirements (e.g.: in this case issues recovering stool)<br><br>The average RSD for triplicate measurements of nanoplastics was 0.88 ± 0.45 %	Microwave acid digesters vary in sample capacity, and consequently the time required to measure a given number of samples varies. After instrument daily checks, ICP-MS measurements take approximately 2 minutes per sample. With our current laboratory infrastructure, from digestion to analysis, approximately 30 samples can be measured in a day.	Both microwave acid digestion and ICP-MS analysis are common and routine analytical devices and procedures for laboratories suited to trace metal analysis
IMC	In the context of proteins detected on single cells with antibodies tagged to a metal, the LOD is ≥400 copies/µm², however, based on the titration experiment in this study, one Pd-NP is estimated to be one dual count per pixel	Pixels are displayed with their x and y position in space on a tissue slide, so the position of every pixel is known and is 1µm^2	None<br>IMC directly measures metal isotope counts based on the time the metal hits the detector. This is extremely precise because this time is verified daily in the quality control methods.	High<br><br>IMC is highly reproducible because metals are more stable than fluorophores and do not degrade over time. Also, laser ablation is quality controlled daily to ensure correct ablation and sensitivity.	2 hours per 1 mm^2 tissue area acquired. We limit our ROI size to between 1-2 mm^2. The short exposure experiment in this study had 24 ROI acquired and an acquisition time of two days	There are 3 facilities in Switzerland that offer Hyperion services.

Table S13: Characteristics of nanoplastic suspension.

Hydrodynamic diameter [nm]	Polydispersity Index	Zeta-potential [mV]	Pd content per mass of NP [% w/w]
210	0.1	-49.8	0.295 ± 0.009

Table S14: Mouse IDs and treatment details for all 3 experiments.

**Acute exposure experiment**			
**Mouse ID**	**Sex**	**Genotype**	**Treatment / Details**
28692	m	C57Bl/6J	10 min
28693	m	C57Bl/6J	10 min
28714	m	C57Bl/6J	30 min
28715	m	C57Bl/6J	30 min
28716	m	C57Bl/6J	60 min
28717	m	C57Bl/6J	60 min
28667	m	C57Bl/6J	120 min
28668	m	C57Bl/6J	120 min
28670	m	C57Bl/6J	240 min
28671	m	C57Bl/6J	240 min
**Subacute exposure experiment**			
**Mouse ID**	**Sex**	**Genotype**	**Treatment / Details**
28327	m	C57Bl/6J	Group 1 - Control
28328	m	C57Bl/6J	Group 1 - Control
28329	m	C57Bl/6J	Group 1 - Control
28330	m	C57Bl/6J	Group 3 - DSS
28331	m	C57Bl/6J	Group 3 - DSS
28332	m	C57Bl/6J	Group 3 - DSS
28333	m	C57Bl/6J	Group 4 – Pd-NPs + DSS
28334	m	C57Bl/6J	Group 4 – Pd-NPs + DSS
28336	m	C57Bl/6J	Group 2 – Pd-NPs
28337	m	C57Bl/6J	Group 2 – Pd-NPs
28338	m	C57Bl/6J	Group 2 – Pd-NPs
**Subchronic exposure experiment**			
**Mouse ID**	**Sex**	**Genotype**	**Treatment / Details**
29193	m	C57BL/6J	healthy wildtype + Pd-NPs (dropout)
29194	m	C57BL/6J	healthy wildtype + Pd-NPs
29199	m	C57BL/6J	healthy wildtype + Pd-NPs
29200	m	APCmin+	diseased APCmin+ + Pd-NPs
29206	m	APCmin+	diseased APCmin+ + Pd-NPs
29207	m	APCmin+	diseased APCmin+ + Pd-NPs

Table S15: ARRIVE checklist on essential (1-10) and recommended (11-21) set of items for reporting animal experiments. Supplementary notes and details not mentioned in the main manuscript text or information of where to find the respective information in the main manuscript and supplementary material.

Item			Recommendation	Section/line, additional comment
**Study design**	1		For each experiment, provide brief details of study design including:	
		a.	The groups being compared, including control groups. If no control group has been used, the rationale should be stated.	see Methods section > Animal Experiments (line 481 ff) and Figure 1
		b.	The experimental unit (e.g. a single animal, litter, or cage of animals)	single animal (or cage in case of feces)
**Sample size**	2	a.	Specify the exact number of experimental units allocated to each group, and the total number in each experiment. Also indicate the total number of animals used	see Methods section > Animal Experiments (line 481 ff) and Figure 1, Table S9
		b.	Explain how the sample size was decided. Provide details of any a priori sample size calculation, if done.	Not applicable.<br>The main aim of the study was to show the feasibility of different analytical methods (see also Discussion line 437 ff)
**Inclusion and exclusion criteria**	3	a.	Describe any criteria used for including and excluding animals (or experimental units) during the experiment, and data points during the analysis. Specify if these criteria were established a priori. If no criteria were set, state this explicitly.	Not applicable.<br>All measured data are shown (see Supplementary Material and Data).
		b.	For each experimental group, report any animals, experimental units or data points not included in the analysis and explain why. If there were no exclusions, state so.	For the subchronic exposure experiment one mouse in the wildtype group dropped out, due to complications during gavage. No analyzed data points were excluded and all measured data are shown in the Supplementary Material and Data.
		c.	For each analysis, report the exact value of n in each experimental group.	Methods > Animal Experiments (line 481 ff), Figure 1, Table S9 and respective figure caption
**Randomisation**	4	a.	State whether randomisation was used to allocate experimental units to control and treatment groups. If done, provide the method used to generate the randomisation sequence.	Methods > Animal Experiments (line 491 ff)
		b.	Describe the strategy used to minimise potential confounders such as the order of treatments and measurements, or animal/cage location. If confounders were not controlled, state this explicitly.	Not applicable/ No control of confounders.<br>The main aim of the study was to show the feasibility of different analytical methods.
**Blinding**	5		Describe who was aware of the group allocation at the different stages of the experiment (during the allocation, the conduct of the experiment, the outcome assessment, and the data analysis).	No blinding of group allocation was done as mouse experiment and sample analysis were performed at different institutions. Blinding of the analytical process was not deemed necessary, as the applied machine-based analytical methods operate in an unbiased manner, independent of sample identity or experimental conditions. Measurements were performed at equal conditions for all samples of an experiment.
**Outcomes measures**	6	a.	Clearly define all outcome measures assessed (e.g. cell death, molecular markers, or behavioural changes)	see Methods; Pd concentration /sample and respective recalculation to % daily dose applied (for total organ), spatial location of Pd-doped Pd-NPs in tissue.
		b.	For hypothesis-testing studies, specify the primary outcome measure, i.e. the outcome measure that was used to determine the sample size.	Not applicable.<br>The main aim of the study was to show the feasibility of different analytical methods.
**Statistical methods**	7	a.	Provide details of the statistical methods used for each analysis, including software used.	Not applicable.<br>Due to the small sample size (feasibility study) no statistical analysis was performed.
		b.	Describe any methods used to assess whether the data met the assumptions of the statistical approach, and what was done if the assumptions were not met.	Not applicable.
**Experimental animals**	8	a.	Provide species-appropriate details of the animals used, including species, strain and substrain, sex, age or developmental stage, and, if relevant, weight.	see Methods > Animal Experiments (line 481 ff) and Table S9

		b. Provide further relevant information on the provenance of animals, health/immune status, genetic modification status, genotype, and any previous procedures.	see Methods > Animal Experiments (line 481 ff) and Table S9
**Experimental procedures**	9	For each experimental group, including controls, describe the procedures in enough detail to allow others to replicate them, including:	
		a. What was done, how it was done and what was used.	see Methods > Animal Experiments (line 481 ff), and Figure 1
		b. When and how often.	see Methods > Animal Experiments (line 481 ff), and Figure 1
		c. Where (including detail of any acclimatisation periods).	Experiments were performed in the animal facility of the Medical University Vienna. Animals were bread in house and after randomization at weaning (3 weeks), mice were acclimatized in the respective experimental rooms until the start of the experiment (8-12 weeks of age).
		d. Why (provide rationale for procedures).	Introduction (line 115 ff) and Results (beginning of each experimental paragraph)
**Results**	10	For each experiment conducted, including independent replications, report:	
		a. Summary/descriptive statistics for each experimental group, with a measure of variability where applicable (e.g. mean and SD, or median and range).	Not applicable.<br>Individual measurements > see Supplementary Tables
		b. If applicable, the effect size with a confidence interval.	Not applicable.<br>Individual measurements
**Abstract**	11	Provide an accurate summary of the research objectives, animal species, strain, and sex, key methods, principal findings, and study conclusions.	Only partially in Abstract as this was not the main focus of this paper. See also Introduction, Methods, Results and Discussion sections.
**Background**	12	a. Include sufficient scientific background to understand the rationale and context for the study, and explain the experimental approach.	Introduction (line 115 ff), Methods > Animal Experiments, Figure 1, Results (introduction to each experiment)
		b. Explain how the animal species and model used address the scientific objectives and, where appropriate, the relevance to human biology.	Introduction (line 115 ff)
**Objectives**	13	Clearly describe the research question, research objectives and, where appropriate, specific hypotheses being tested.	Introduction (line 115 ff)
**Ethical statement**	14	Provide the name of the ethical review committee or equivalent that has approved the use of animals in this study, and any relevant licence or protocol numbers (if applicable). If ethical approval was not sought or granted, provide a justification.	Methods > Animal Experiments (line 545 ff)
**Housing and husbandry**	15	Provide details of housing and husbandry conditions, including any environmental enrichment.	Methods > Animal Experiments (line 547 ff)<br>Breeding colonies were kept separated from experimental rooms and under specific pathogen free (SPF) conditions in individual ventilated cages (IVCs). Mice in the experimental room were kept under optimized hygiene conditions (OHB) in type 3 filter hood cages at maximum 5 individuals per cage. Mice were kept under standardized conditions (room temperature 23 ± 2°C, humidity 55 ± 10%, at 12h dark/light cycle) on poplar bedding (LASbedding PG2 bedding, LASvendi). For environmental enrichment mice received shredded paper strips as nesting material, wood gnawing blocks, a wooden tunnel and a house (red translucent plastic). Mice were fed a standardized rodent diet (LASQCdiet Rod16, Altromin, Germany) and provided drinking water *ad libitum*. All materials were autoclaved before use and cages exchanged at least once a week. Mice were tested negative for all FELASA- relevant murine infectious agents at all quarterly health inspections.
**Animal care and monitoring**	16	a. Describe any interventions or steps taken in the experimental protocols to reduce pain, suffering and distress.	5-9 weeks acclimatization period to experimental room, with handling training (subacute and subchronic experiments) 2 weeks prior to gavage start. DSS treated mice

				received additional pre-wetted food at the cage bottom. Maximum duration on metal grid (for feces collection) was 24 h followed by at least 24 h on regular bedding material.
		b.	Report any expected or unexpected adverse events.	For the subchronic exposure experiment one mouse in the wildtype group had complications during gavage and needed to be sacrificed (known adverse event for this gavage method).
		c.	Describe the humane endpoints established for the study, the signs that were monitored and the frequency of monitoring. If the study did not have humane endpoints, state this.	Human endpoints: > 20% body weight loss, reduced mobility/apathy, respiratory distress, severe pain, anemia (APCmin+), bad health status as defined by scoring sheet;<br>Mice were visually inspected at least once daily and monitoring was performed daily on weekdays (for DSS cohorts or if indicated by health status also on weekends).<br>Monitoring parameters: weight, activity, signs of pain (hunched posture, coat), diarrhea, rectal bleeding (DSS and APCmin+), anemia (APCmin+)
**Interpretation/ scientific implications**	17	a.	Interpret the results, taking into account the study objectives and hypotheses, current theory and other relevant studies in the literature	see Discussion
		b.	Comment on the study limitations including potential sources of bias, limitations of the animal model, and imprecision associated with the results.	see Discussion (line 437 ff)
**Generalisability/ translation**	18		Comment on whether, and how, the findings of this study are likely to generalize to other species or experimental conditions, including any relevance to human biology (where appropriate)	see Discussion
**Protocol registration**	19		Provide a statement indicating whether a protocol (including the research question, key design features, and analysis plan) was prepared before the study, and if and where this protocol was registered.	The study protocol was not pre-registered.
**Data access**	20		Provide a statement describing if and where study data are available.	see Data Availability Statement (line 679 ff)
**Declaration of interests**	21	a.	Declare any potential conflicts of interest, including financial and non-financial. If none exist, this should be stated.	see Competing Interests Statement (line 836 ff)
		b.	List all funding sources (including grant identifier) and the role of the funder(s) in the design, analysis and reporting of the study.	see Acknowledgement Section (line 810 ff)

Table S16: Summary of ICP-MS instrument and sample LOD/LOQ values across the three experiments.

	LOD	LOQ
**Acute exposure experiment**		
Instrumental (µg/L)	0.014	0.043
Blood sample (ng)	0.14	0.43
Organ sample (ng)	0.42	1.29
Feces sample (ng)	0.19	0.56
**Subacute exposure experiment**		
Instrumental (µg/L)	0.019	0.056
Blood sample (ng)	0.24	0.70
Organ sample (ng)	1.43	4.20
Feces sample (ng)	0.43	1.26
**Subchronic exposure experiment**		
Instrumental (µg/L)	0.019	0.053
Blood sample (ng)	0.30	0.88
Organ sample (ng)	0.65	1.89
Feces sample (ng)	0.19	0.56

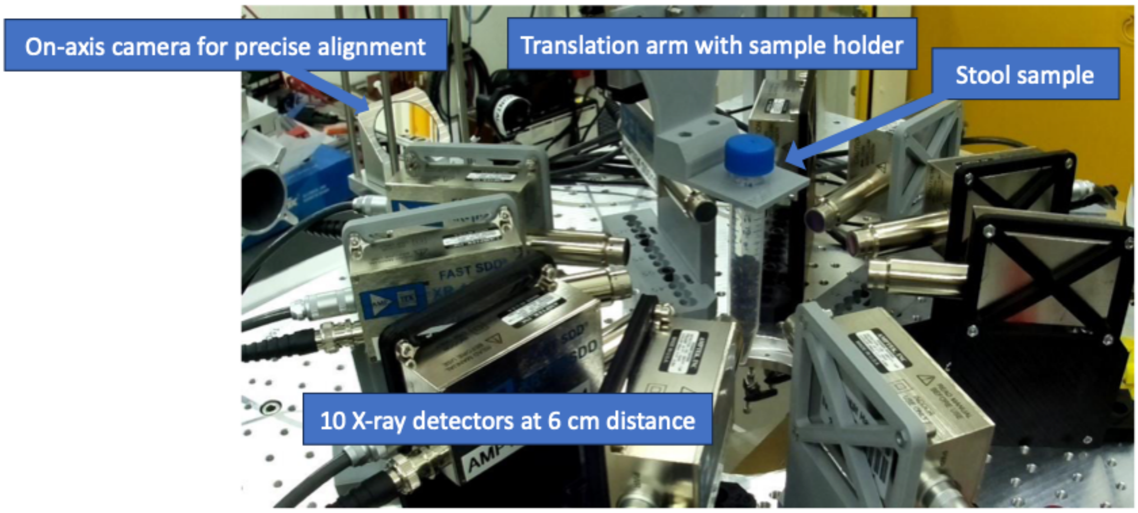

Figure S5: Picture of the custom-built experimental setup at the P21.1 beamline at the PETRA III synchrotron (DESY, Hamburg, Germany) consisting of 10 radiation detectors on a platform which allows scanning of samples through the incident X-ray beam. The on-axis camera on the left side allows for precise alignment of the samples and shows the direction of the incident X-ray beam.

Table S17: Summary of the main beam parameters used in the XFI measurement campaigns.

Experiment	Incident energy [keV]	Photon flux [ph/s/mm^2]	Scan time per pixel [s]
Acute	53.03	1.1 x 10^{11}	10 - 60
Subacute	53.15	2.4 x 10^{10}	20
Subchronic	53.06	5.5 x 10^{10}	30 - 60

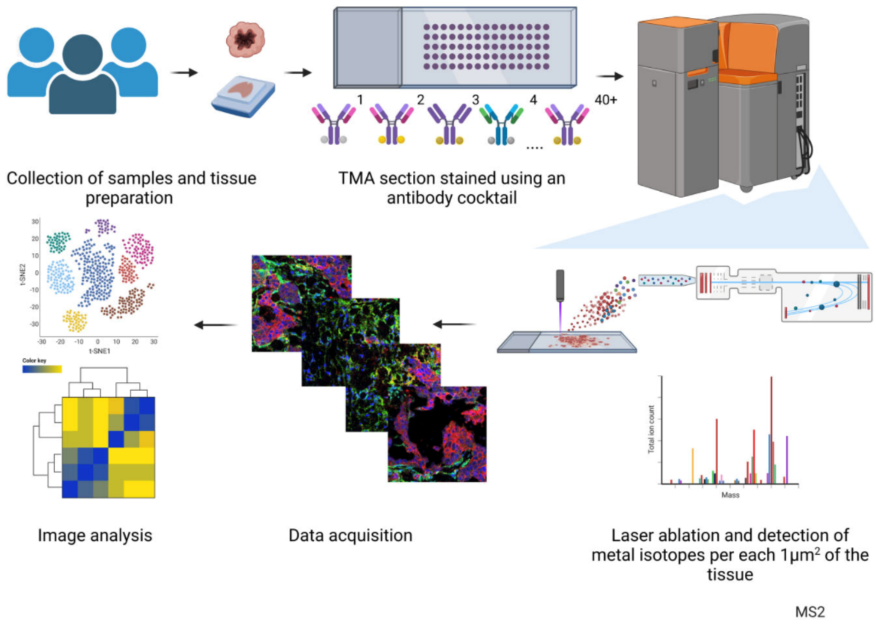

Figure S6: Overview of how IMC is typically performed. Patients (or mice) provide tissue that is formalin fixed paraffin embedded (FFPE) and stained with antibodies bound to heavy metals (in this study, mice were gavaged with Pd-NPs) and then the tissues are laser ablated directly or punched into a tissue micro array (TMA) and then the cores are ablated.[1]

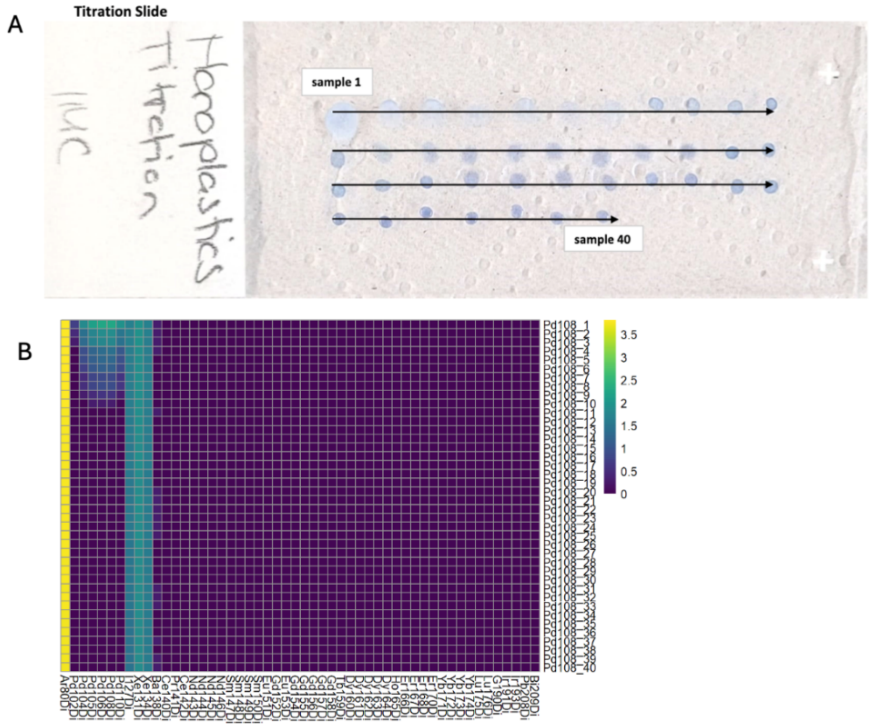

Figure S7: Pd-NPs titration experiment. A) Slide setup with titration dilution spots. B) Heatmap of all 40 dilutions (spots) (y-axis) and all IMC mass windows open (x-axis).

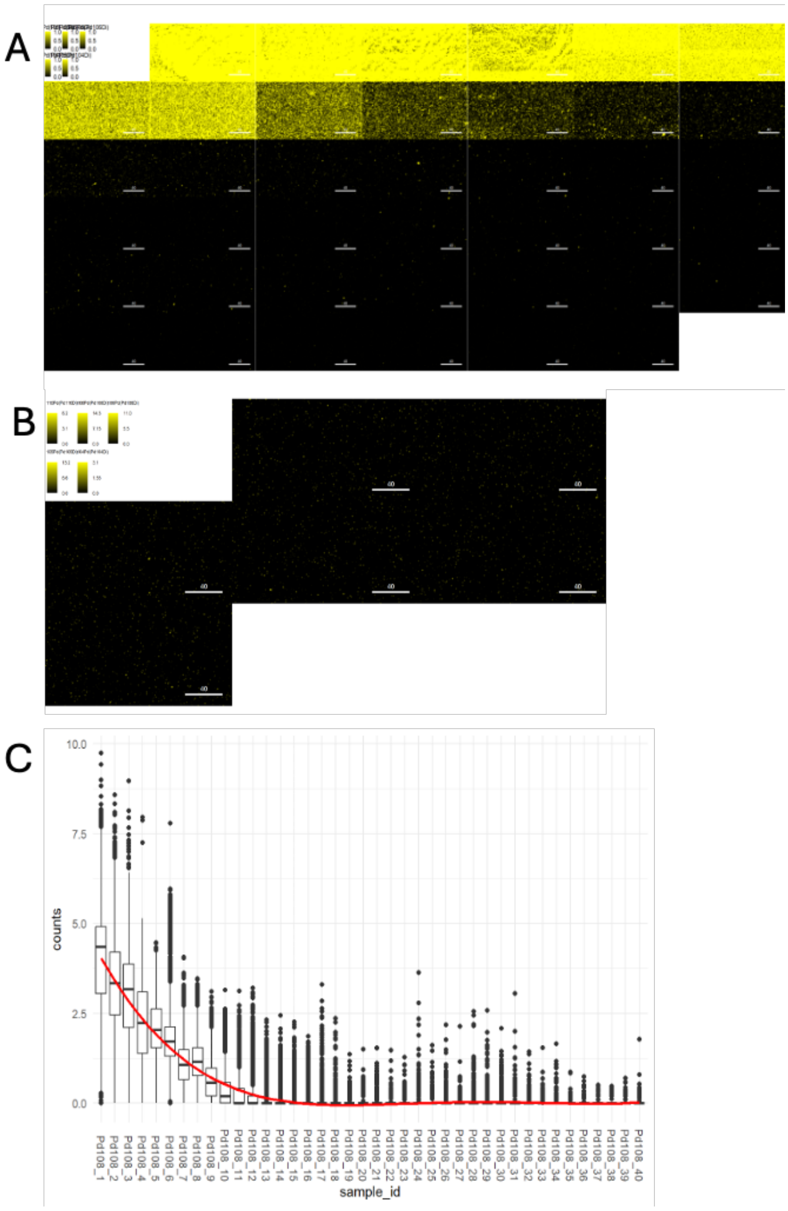

Figure S8: IMC ROI images from the Pd-NP titration experiment. A) The ROI images of Pd in yellow in each of the 40 spots diluted in a series 1:1 with all Pd isotopes shown in yellow B) Final 6 ROIs zoomed in. C) Dual counts of palladium per pixel (y-axis) per ROI (x-axis) in each of the 40 Pd-NP dilution. The box plots display the first (upper) and third (lower) quartiles around the median, with the black line in the middle of the box indicating the actual median value. All points that fall outside this range are indicated as dots above and below the boxes.

References:

(1) Milosevic, V. Different Approaches to Imaging Mass Cytometry Data Analysis. *Bioinforma. Adv.* **2023**, *3* (1), vbad046. https://doi.org/10.1093/bioadv/vbad046.
